# Prophylactic efficacy of orally administered *Bacillus* poly-γ-glutamic acid, a non-LPS TLR4 ligand, against norovirus infection in mice

**DOI:** 10.1038/s41598-018-26935-y

**Published:** 2018-06-06

**Authors:** Wooseong Lee, Minwoo Kim, Seung-Hoon Lee, Hae-Gwang Jung, Jong-Won Oh

**Affiliations:** Department of Biotechnology, Yonsei University, Seoul, 03722 Korea

## Abstract

Poly-gamma-glutamic acid (γ-PGA), an extracellular biopolymer produced by *Bacillus* sp., is a non-canonical toll-like receptor 4 (TLR4) agonist. Here we show its antiviral efficacy against noroviruses. γ-PGA with a molecular mass of 2,000-kDa limited murine norovirus (MNV) replication in the macrophage cell line RAW264.7 by inducing interferon (IFN)-β and conferred resistance to viral infection-induced cell death. Additionally, γ-PGA interfered with viral entry into cells. The potent antiviral state mounted by γ-PGA was not attributed to the upregulation of TLR4 or TLR3, a sensor known to recognize norovirus RNA. γ-PGA sensing by TLR4 required the two TLR4-associated accessory factors MD2 and CD14. In *ex vivo* cultures of mouse ileum, γ-PGA selectively increased the expression of IFN-β in villi. In contrast, IFN-β induction was negligible in the ileal Peyer’s patches (PPs) where its expression was primarily induced by the replication of MNV. Oral administration of γ-PGA, which increased serum IFN-β levels without inducing proinflammatory cytokines, reduced MNV loads in the ileum with PPs and mesenteric lymph nodes in mice. Our results disclose a γ-PGA-mediated non-conventional TLR4 signaling in the ileum, highlighting the potential use of γ-PGA as a prophylactic antiviral agent against noroviruses.

## Introduction

Toll-like receptor (TLR) 4 is a pattern-recognition receptor (PRR) that recognizes lipopolysaccharide (LPS) from Gram-negative bacteria as a pathogen-associated molecular pattern (PAMP). TLR4 signaling activates the MyD88 (myeloid differentiation primary-response protein 88)- and TRIF (TIR domain-containing adaptor protein-inducing interferon-β)-dependent pathways to induce the production of proinflammatory cytokines and interferons (IFNs), respectively^1–4^. Since the discovery of TLR4 as a receptor for LPS, several non-LPS TLR4 ligands, which are mostly derived from microbes, have been identified and their roles in TLR4-mediated innate immune responses have been proposed^5–8^. During the onset of intestinal inflammation in response to PAMPs from colon and possibly non-LPS TLR4 ligands from diet, TLR4 signaling is likely to play an important role in intestinal epithelial cells (IECs) and immune cells, including, macrophages and dendritic cells. IFN and/or inflammatory cytokines produced in the intestine upon stimulation by these PAMPs would systemically act on virus-infected cells locally and/or far away from sites where IFN signaling was initially induced. However, TLR4 signaling in the small intestine is not well characterized^9^.

Noroviruses are positive-sense, single-stranded RNA viruses belonging to the *Caliciviridae* family^10^. Human norovirus (HuNoV) is transmitted via the fecal-oral route and propagates in the gastrointestinal (GI) tract^11^. It is the most common cause of non-bacterial acute outbreaks of gastroenteritis worldwide across all age groups^12^. Norovirus has become a global health burden due to its high-sustained viability in environment, high risk of infection with fewer than 100 particles, possibility of causing chronic infection in immune deficient hosts as well as the elderly and infants, and emergence of novel norovirus strains^13–16^. Despite the breadth of potential antiviral drugs tested *in vitro* using a HuNoV replicon culture system^17^, limited studies have validated the efficacy of these drugs *in vivo*^18–20^. There are no reports on safe, prophylactic antiviral drugs that could prevent the onset of acute gastroenteritis in immunocompromised hosts. In the absence of approved vaccines and specific antiviral therapies for HuNoV^18,21,22^, development of prophylactic or therapeutic measures against HuNoV has become the need of the hour.

Poly-γ-glutamic acid (γ-PGA) is an anionic polypeptide synthesized and secreted by *Bacillus* species. In γ-PGA, d- and/or l-glutamate is polymerized via γ-amide linkages formed between the α-amino and γ-carboxylic acid functional groups^23^. We had previously reported the activation of TLR4 signaling pathway by γ-PGA, leading to the production of type I interferon (IFN) (α and β)^6^. *In vitro*, γ-PGA displays antiviral activity against severe acute respiratory syndrome coronavirus and hepatitis C virus^6^. In norovirus-infected cells, IFN-α/β production is initiated by the sensing of viral RNA genome via the cytoplasmic PRR MDA5 (melanoma differentiation-associated gene 5) and TLR3, followed by phosphorylation of IFN regulatory factor 3 (IRF-3) through two independent pathways^24^. This type I IFN signaling plays a major role in clearing mouse norovirus^24–26^ and HuNoV^27,28^.

γ-PGA is a major ingredient of “natto”, a Japanese traditional fermented food, and “Chungkookjang” a Korean fermented seasoning, both made from soybeans^6^ and used for dietary consumption. In the present study, we investigated whether γ-PGA, a non-LPS TLR4 ligand, could activate TLR4 signaling when administered orally. Our results demonstrate the induction of IFN-β by peroral administration of γ-PGA without the production of proinflammatory cytokines, thereby establishing an antiviral state against mouse norovirus. We provide evidence of *in vivo* efficacy of γ-PGA in limiting norovirus infection, highlighting the potential of γ-PGA as a prophylactic antiviral agent.

## Results

### Induction of IFN-β expression by γ-PGA inhibits mouse norovirus replication and viral infection-induced cell death

**Figure 1:**
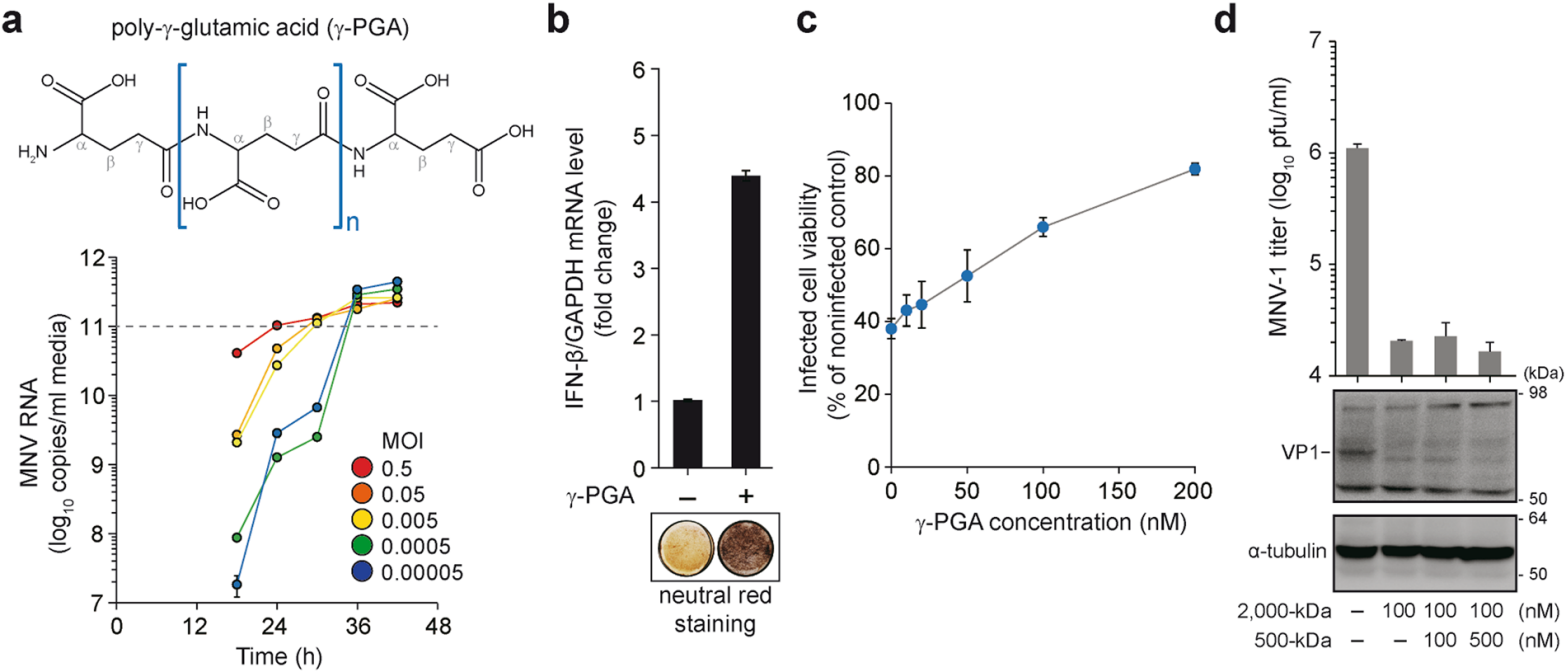
Inhibition of norovirus infection-induced apoptosis by the TLR4 ligand γ-PGA. (**a**) Molecular structure of γ-PGA with γ-linkage (top). RT-qPCR quantification of viral genome titers in the culture media from RAW264.7 cells infected with increasing MOI of murine norovirus 1 (MNV-1). Approximately 60–70% cells were dead when the viral genome titer reached to a titer of 1 × 10^11^ copies/ml media denoted by the dotted line. (**b**) RT-qPCR quantification of the expression of IFN-β mRNA in RAW264.7 cells treated with 2,000-kDa γ-PGA (100 nM) for 18 h. Shown is the GAPDH-normalized IFN-β mRNA level relative to that in mock-treated cells. Presented below each bar are representative images of RAW264.7 cells stained with neutral red, 30 h post-infection with MNV-1 at an MOI of 0.005. (**c**) RAW264.7 cells infected with MNV-1 (MOI of 0.005) were treated with the indicated doses of 2,000-kDa γ-PGA. Cell viability was quantified by the MTS assay at 36 h post-infection. (**d**) MNV-1 (MOI of 0.005)-infected RAW264.7 cells were treated with the indicated concentrations of 2,000-kDa γ-PGA with or without 500-kDa γ-PGA. Viral genome and VP1 levels were analyzed 30 h later by RT-qPCR and immunoblotting (IB), respectively. In (**c**,**d**), data are mean ± s.d.

HuNoV infection causes epithelial apoptosis and downregulates the level of tight junction proteins involved in sealing, leading to diarrhea^29^. Comparably, murine norovirus (MNV) infection also induces apoptosis in RAW264.7 cells^30^. In the absence of a robust cell culture system for HuNoV^31^, we used murine norovirus 1 (MNV-1), as a surrogate model for HuNoV, to test for the inhibition of virus-induced cell death by γ-PGA. We first determined the range of multiplicity of infection (MOI) of MNV in which viral infection-induced apoptosis is triggered during the course of viral infection. As shown in Fig. 1a, infection of RAW264.7 cells with MNV at an MOI of ≥0.005 produced ≥1 × 10^11^ viral RNA copies/ml of media within 30 h. With an MOI ≤0.0005, there was a >10-fold reduction in viral RNA levels, delaying the progress of cell death within 2 days. Accordingly, we used an MOI of 0.005 for infecting cells to assess the impact of γ-PGA. As shown in Fig. 1b, treatment with 2,000-kDa γ-PGA significantly inhibited MNV infection-induced cell death as visualized by live cell staining, with a 4.4-fold increase in IFN-β mRNA expression than in non-treated control cells. γ-PGA dose-dependently prevented virus-induced cell death resulting in increased cell viability (from approximately 40% to 80% at 200 nM; Fig. 1c), demonstrating the potent antiviral activity of this non-canonical TLR4 ligand against MNV.

Furthermore, treatment with 100 nM γ-PGA resulted in a 98% decrease in plaque titer and significantly reduced the level of VP1, a viral structural protein (Fig. 1d). The antiviral potency of 2,000-kDa γ-PGA was comparable to that of 17-DMAG (Supplementary Fig. 1, mean 50% effective concentration of 17-DMAG: 35 nM), an Hsp90 inhibitor that has been recently shown to be effective in controlling MNV infection^32^. The antiviral activity of 2,000-kDa γ-PGA was not attenuated by co-treatment with 500-kDa γ-PGA at 100 nM or 500 nM, suggesting the importance of molecular mass or a specific structure formed by 2,000-kDa γ-PGA for the activation of TLR4 signaling.

### γ-PGA inhibits norovirus replication and entry without upregulating TLR expression

**Figure 2:**
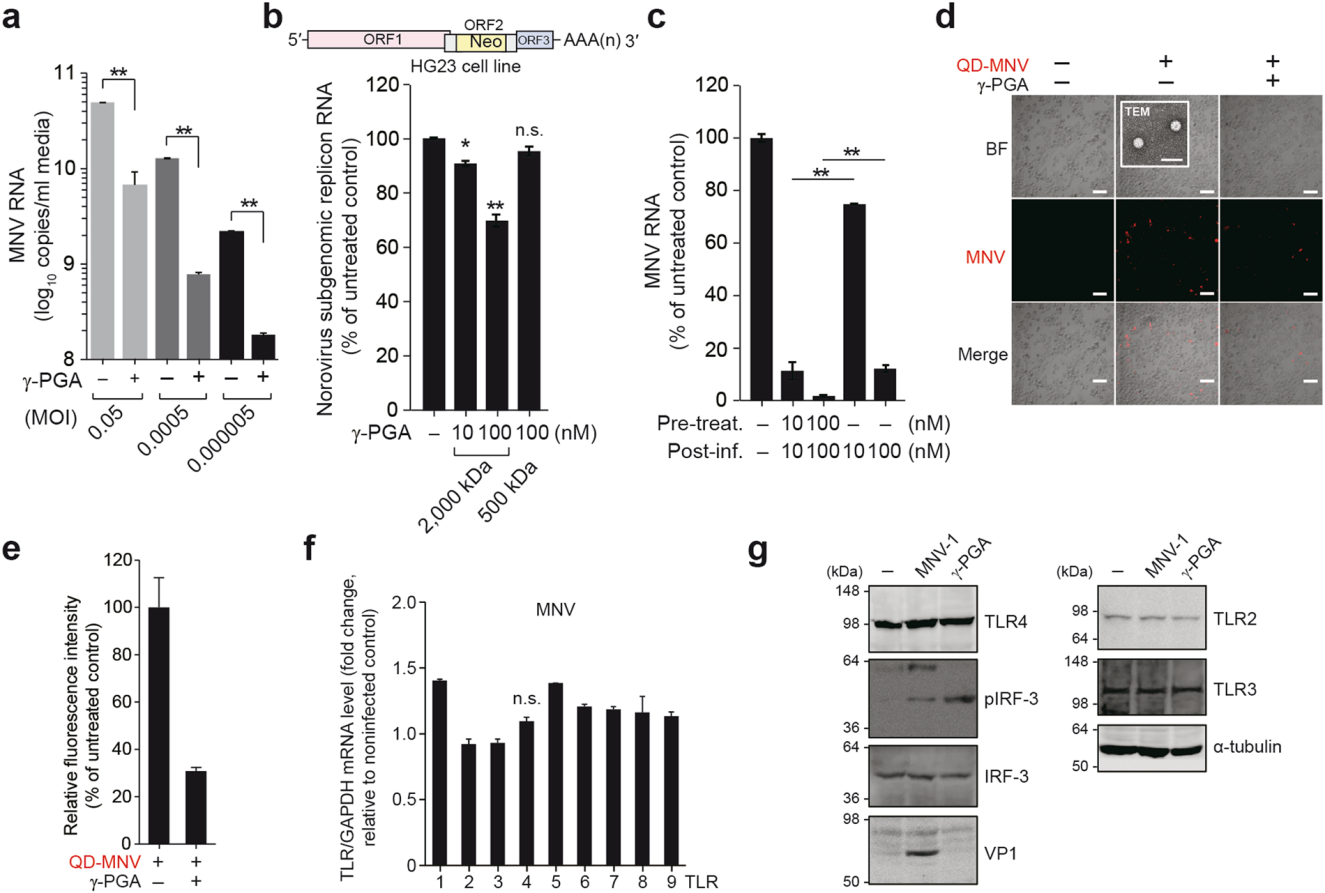
Analysis of the antiviral activity of γ-PGA against mouse and human noroviruses. (**a**) RAW264.7 cells infected with the indicated MOI of MNV-1 were treated with 100 nM 2,000-kDa γ-PGA. Viral genome copy number and infectious virus titer in the culture media were determined by RT-qPCR at 40 h post-infection. (**b**) The HG23 cell line harboring a Norwalk virus sub-genomic replicon was transfected with plasmids expressing TLR4, MD2, and CD14 individually. After incubation for 6 h, transfected cells were treated with γ-PGA (100 nM) for 2 days, prior to RT-qPCR quantification of replicon RNA titer two days later. Neo, neomycin phosphotransferase-coding gene conferring resistance to G418. (**c**) RAW264.7 cells were pre-treated for 6 h before MNV-1 infection or treated after the viral infection with increasing concentrations of 2,000-kDa γ-PGA. Viral genome titer was determined at 30 h post-infection. (**d**,**e**) Inhibition of MNV-1 entry by γ-PGA. Q-dot (QD)-conjugated MNV-1, which was pre-incubated with 2,000-kDa γ-PGA (100 nM) for 30 min, was applied to RAW264.7 cells. Two hours after adsorption, viral entry was quantified by fluorescence microscopy (**d**), and the fluorescence intensity signals were quantified by image analysis using ImageJ software (**e**). BF, bright field image. Scale bar, 50 μm. Shown as an inset in (**d**) is the transmission electron micrograph of purified MNV particles negatively stained with uranyl acetate (Scale bar, 100 nM). (**f**) Expression of TLR1-9 in RAW264.7 cells infected with MNV-1 (MOI of 0.005). Shown are the GAPDH-normalized TLR expression levels relative to those in mock-treated cells, analyzed by RT-qPCR at 24 h post-infection. Results are from two independent experiments, each analyzed by triplicate assay. (**g**) Immunoblot assay representing the activation of IRF-3 by γ-PGA (100 nM) and MNV-1 infection (MOI of 0.005) without affecting TLR2, TLR3, and TLR4 expression levels. pIRF-3, phosphorylated IRF-3. In all panels, data are mean ± s.d. Statistical significance of differences between groups was determined via two-tailed unpaired Student’s *t*-test. *P < 0.05; **P < 0.01; n.s., not significant.

Inhibition of MNV by 2,000-kDa γ-PGA was further verified by measuring intracellular viral genome titers. γ-PGA reduced viral genome titer by up to 92% in cells infected with a low MOI (0.000005). Its inhibitory effect decreased slightly (86% inhibition) at an MOI of 0.05 (Fig. 2a). Additionally, it led to a 30% reduction in the level of Norwalk virus sub-genomic replicon (derived from a GI strain of HuNoV) at 100 nM. In HG23 cell line, 500-kDa γ-PGA had little impact on the replication of sub-genomic replicon (Fig. 2b). The HG23 replicon cell line derived from the human hepatocellular carcinoma cell line Huh7 normally fails to respond to TLR4 ligands because of the low-level expression of TLR4 expression together with the lack of the expression of its accessory proteins, CD14 and MD2^6^. Thus, the insufficient, transient expression of TLR4 and the associated accessory proteins might have contributed to the relatively lower inhibitory effect. The critical role of these accessory proteins in γ-PGA sensing and TLR4-mediated IFN-β expression was further investigated in human embryonic kidney 293 T cells (HEK293T), which express no endogenous TLR4, MD2, and CD14^33,34^. By confocal microscopy, we found that γ-PGA sensing and intracellular entry occurred upon ectopic expression of both TLR4 and its two known accessory factors (Supplementary Fig. 2a–c). Substantial induction of IFN-β expression and IRF-3 activation were observed only when TLR4, MD2, and CD14 were expressed (Supplementary Fig. 2d). This TLR4-mediated IFN-β induction capability of γ-PGA was also verified in RAW264.7 cells in which RNAi-mediated depletion of TLR4 abrogated IFN-β expression and IRF-3 activation (Supplementary Fig. 2e).

Pre-treatment of cells (6 h before infection) with 2,000-kDa γ-PGA significantly increased its antiviral activity compared to post-infection treatment (1 h after infection) (Fig. 2c). This finding suggests that treatment with γ-PGA predisposes cells to acquire resistance to viral infection that is effective in suppressing MNV propagation. Additionally, the observation raised the possibility of γ-PGA interfering with the entry of MNV. Accordingly, a virus entry assay using Q-dot-conjugated norovirus was conducted. The result indicated the capability of γ-PGA to block the entry of norovirus into RAW264.7 cells (Fig. 2d,e) and explains a relatively lower antiviral activity in HG23 replicon cells than in MNV-infected macrophage cells.

Upon virus infection, upregulation of TLR4 or crosstalk between TLR signaling pathways^35^ might alter the sensitivity of TLR4 to γ-PGA. Alterations in the expression profile of TLRs including TLR4 following norovirus infection was further investigated. TLR1-9 mRNA expression levels were not altered by more than 2-fold in MNV-1-infected RAW264.7 cells (Fig. 2f). Neither norovirus infection nor γ-PGA treatment enhanced TLR4 expression. Immunoblotting analyses detected pIRF-3, indicating that both these stimuli activated TLR4 signaling as evidenced by the activation of IRF-3 (Fig. 2g). Similarly, neither TLR3, a sensor for norovirus dsRNA^24^ nor TLR2, which could be a potential innate immune sensor for not-yet-identified norovirus-derived or induced PAMPs, was upregulated by norovirus infection or γ-PGA. These results suggest that the antiviral activity of γ-PGA is not dependent on the upregulation of TLRs.

### Sensing of γ-PGA by TLR4 triggers induction of IFN-β in the small intestine of mouse

Norovirus consumed with contaminated food and/or water penetrates through intestinal microfold (M) cells and infects its target cells including macrophages and dendritic cells (for MNV)^25^ or B cells (for HuNoV)^36^. After infection, norovirus propagates in these sentinel cells that are highly enriched in Peyer’s patches (PPs) in the ileum, before spreading to lymph nodes^37,38^.

**Figure 3:**
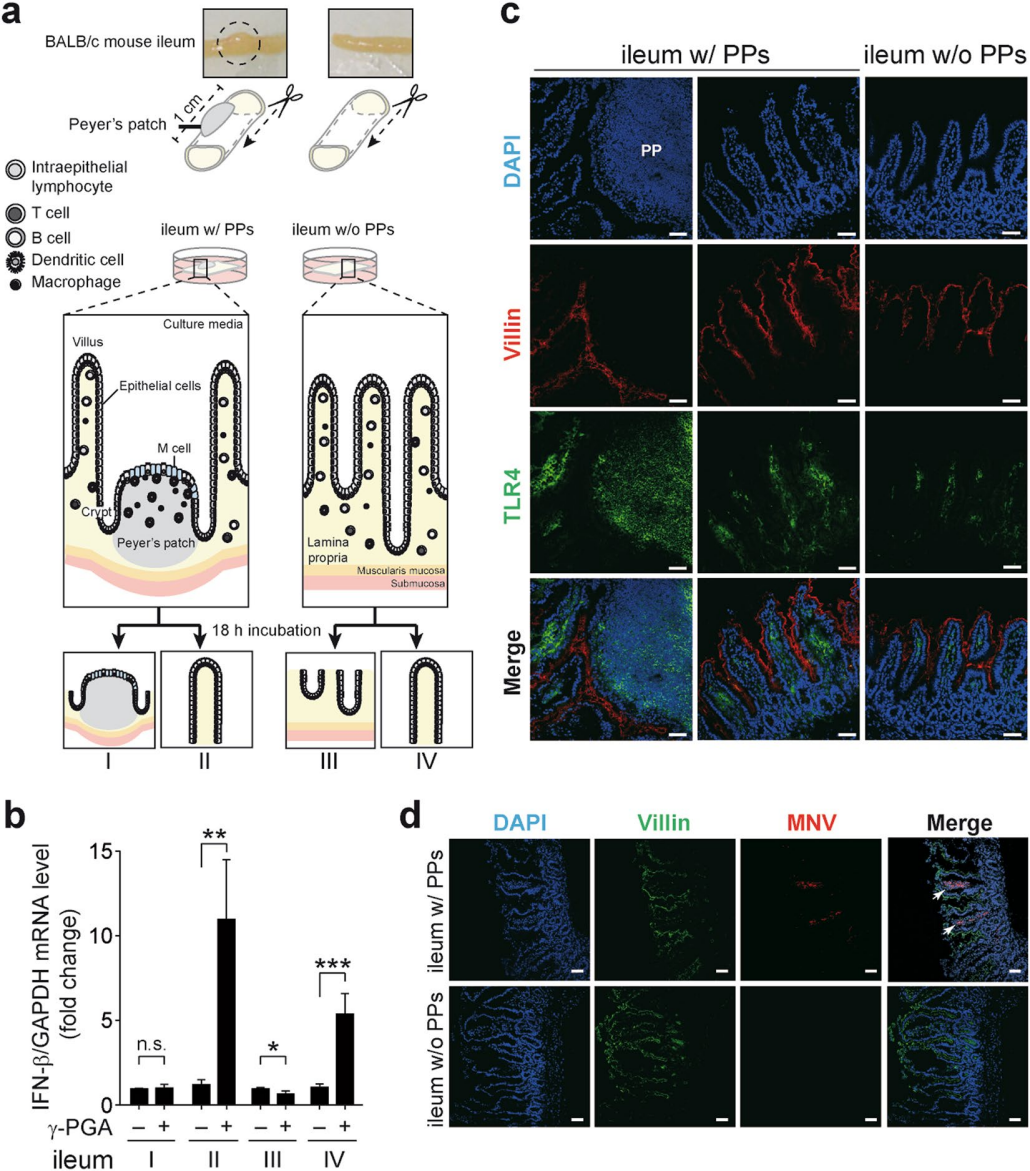
Induction of IFN-β expression by γ-PGA and MNV in mouse ileum. (**a**) Experimental schemes for *ex vivo* stimulation of the ileum with γ-PGA. (**b**) Mouse (*n* = 4) ileum with or without PPs was treated with γ-PGA *ex vivo*. After 18 h incubation, IFN-β mRNA levels in the ileal compartments depicted in (**a**) were determined by RT-qPCR. *P < 0.05; **P < 0.01; ***P < 0.001; n.s., not significant; by two-tailed unpaired Student’s *t*-test. (**c**) Microscopic analysis of TLR4-expressing cells in the ileum with or without PPs. Intestinal microvilli stained with a villin-specific antibody. (**d**) Microscopic analysis of the entry of MNV. Mouse ileum with or without PPs was infected with Qdot fluorophore-tagged MNV *ex vivo* in serum-free DMEM. At 2 h post-infection, cryosection images were taken after extensively washing the infected tissues. Scale bar, 50 μm.

We thus sought to address the question of whether γ-PGA can trigger TLR4 signaling-mediated IFN production in the ileum (composed of epithelial cells plus diverse immune cells in the laminar propria and the PPs) to exert its antiviral activity against norovirus. To this end, distal part of the ileum with or without PPs was retrieved from BALB/c mice. These were treated with γ-PGA in culture dishes for 18 h *ex vivo*. After treatment, the villi released into the culture media and the remaining basal body of the ileum (ileal submucosa and lamina propria) were collected separately and the IFN-β mRNA levels were quantified in these ileal compartments by reverse-transcription quantitative PCR **(**RT-qPCR) (Fig. 3a). Interestingly, we found induction of IFN-β production by γ-PGA only in the villi (compartments II and IV) and not in PPs and basal body of the ileum (compartments III and I) (Fig. 3b). This induction was more substantial in the villi derived from the ileum with PPs (compartment II) than from the ileum without PPs (compartment IV). TLR4-stained cells were more abundant in compartment II than in compartment IV (Fig. 3c). These results suggest that to activate TLR4 signaling, γ-PGA transverses the epithelial cell layer of the villi to reach the laminar propria bearing TLR4-positive macrophages and dendritic cells. Despite the abundance of TLR4-positive cells in PPs, γ-PGA treatment did not significantly induce IFN-β mRNA expression, suggesting non-responsiveness of PPs to γ-PGA by an as-yet-unknown mechanism.

In a parallel experiment, the route of entry of MNV in the ileum was monitored. Fluorescence microscopic analysis of Q-dot-conjugated viruses detected MNV particles in compartment II. No prominent sign of virion entry was detected through the villi at the ileum without PPs (Fig. 3d). These results demonstrate that MNV primarily enters into the ileum bearing PPs through intestinal villi, possibly via M cells or transepithelial dendritic cells. To confirm the entry of MNV through this part of the ileum, viral genome was monitored upon delivering Q-dot-conjugated MNV directly to the small intestine of mouse *ex vivo*. High levels of viral genome was detected in the ileum with PPs [in both compartment I representing PPs and compartment II] 8 h after virus delivery (Supplementary Fig. 3a), in agreement with our results showing MNV particles within villi. Consequently, enhanced IFN-β mRNA levels were detected in both ileal compartments while greater induction was observed in ileal PPs (iPPs) early during virus infection (Supplementary Fig. 3b,c), suggesting that MNV entering through villi reached to the PPs to infect ileal sentinel cells. These results are consistent with the previous findings indicating entry of norovirus through the M cells within the follicle-associated epithelium overlying PPs or in villi to infect macrophages and dendritic cells^37,39,40^.

### Oral administration of γ-PGA induces production of IFN-β but not inflammatory cytokines in mice

**Figure 4:**
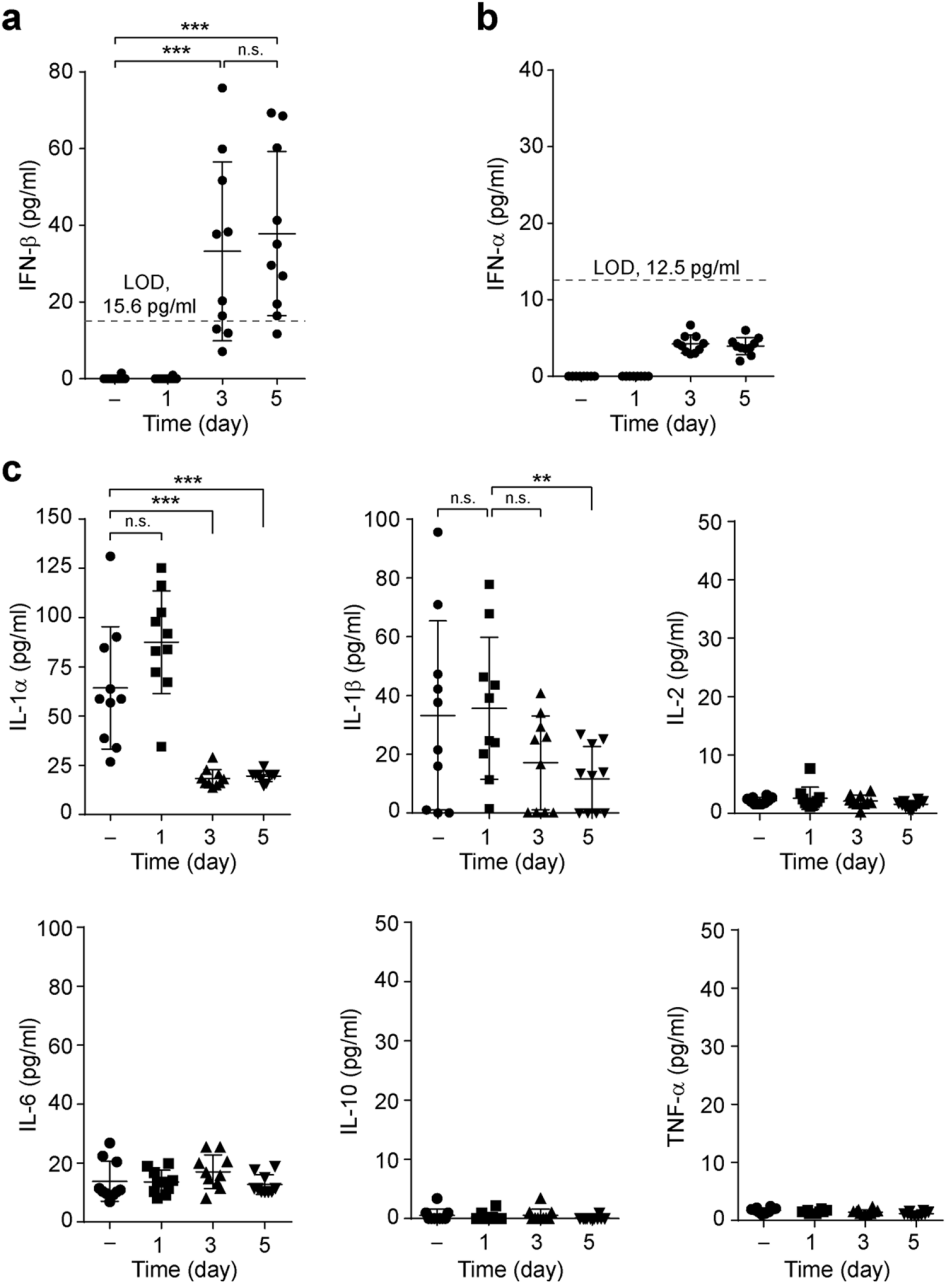
Analysis of cytokines induced by the oral administration of γ-PGA. BALB/c mice (6 weeks old, *n* = 10 per group) were given 2,000-kDa γ-PGA (50 mg/kg of body weight) in 100 µl saline once daily for five days. Blood samples were collected before (day 0) or 4 h after administering γ-PGA on day 1, 3, or 5 to measure titers of IFN-β (**a**), IFN-α (**b**), and other cytokines (**c**) by ELISA. In all panels, data are mean ± s.d. **P < 0.01; ***P < 0.001; n.s., not significant; by two-tailed unpaired Student’s *t*-test. LOD, limit of detection.

IFN-β-inducible activity of γ-PGA was further investigated in mice to ascertain the prophylactic antiviral efficacy of orally delivered γ-PGA. Initial experiments revealed production of both IFN-α and IFN-β at levels below the detection limits of ELISA at 2, 4, 8, 12, and 24 h post-administration of γ-PGA (50 mg/kg body weight per day) to BALB/c mice (Supplementary Fig. 4a,b). These results suggest weak IFN-inducing activity or inefficient delivery of the 2,000-kDa γ-PGA in an intact conformation to the ileum (Fig. 3b,c). When γ-PGA (50 mg/kg body weight) was administered to mice, once a day for five days, serum IFN-β level increased starting from day 3 (Fig. 4a). Induction of IFN-α was negligible (Fig. 4b and Supplementary Fig. 4b). Production of IFN-β was induced by 2,000-kDa γ-PGA but not by 500-kDa γ-PGA (Supplementary Fig. 4c,d), suggesting the importance of its structural entity in activating TLR4 signaling. Furthermore, IFN-β levels (mean values) neither decreased nor increased from day 3 to day 5, indicating that multiple administrations of γ-PGA do not induce TLR4 tolerance to blunt the responsiveness to repetitive challenges with γ-PGA.

Among the other cytokines analyzed, no significant change was detected in the levels of IL-2, IL-6, IL-10, and TNF-α (Fig. 4c). However, significant decrease was seen in the level of IL-1α and IL-1β at day 3 and 5, respectively. Level of IL-1α remained downregulated until day 5 indicating inverse correlation of its expression with IFN-β. These results are consistent with recent studies suggesting a negative regulatory role of IFN-β in the maturation of IL-1α/β^41,42^. Type II interferon (IFN-γ) produced primarily by activated T cells was not detected in all cases. Taken together, our results show that unlike LPS, peroral administration of 2,000-kDa γ-PGA selectively induces IFN-β but not inflammatory cytokines.

### *In vivo* antiviral efficacy of γ-PGA against mouse norovirus

**Figure 5:**
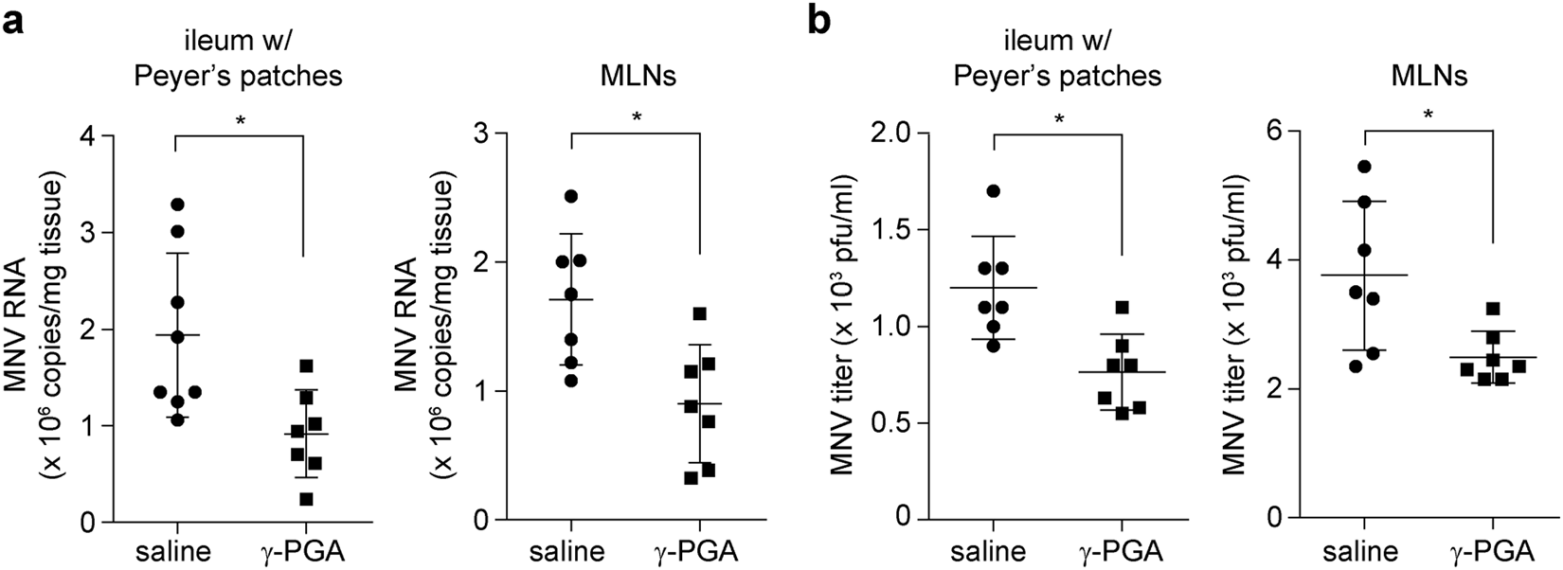
γ-PGA inhibits MNV-1 in mice. BALB/c mice (6 weeks old, *n* = 7–8 per group) were administered with 2,000-kDa γ-PGA as described in Fig. 4 for 5 days. Twelve hours after the last γ-PGA administration, mice were inoculated with MNV-1 (1 × 10^7^ PFU in 100 µl saline) perorally and given γ-PGA once daily for another two days. Viral genome and infectious virus titers in the ileum with PPs and the MLNs were determined by RT-qPCR (**a**) and plaque-forming assay (**b**), respectively. In all panels, data are mean ± s.d. *P < 0.05 by two-tailed unpaired Student’s *t*-test.

To test the antiviral activity of γ-PGA *in vivo*, MNV-1 (1 × 10^7^ PFU per mouse) was orally inoculated into BALB/c mice that received γ-PGA (50 mg/kg body weight) prior to infection once daily for five days to induce IFN-β expression. Two days after MNV inoculation, viral load in the ileum with PPs and mesenteric lymph nodes (MLNs) were quantified using RT-qPCR and plaque-forming assay. γ-PGA administration resulted in 47% and 53% reduction of MNV-1 genome titer in the ileum with PPs and MLNs, respectively (Fig. 5a). Plaque-formation assay revealed 36% and 33% reduction of MNV titer in the ileum with PPs and MLNs, respectively (Fig. 5b). These results indicate that peroral administration of 2,000-kDa γ-PGA effectively suppresses MNV propagation in mouse ileum and MLNs, indicating its prophylactic antiviral efficacy *in vivo*.

### Effect of norovirus infection on TLR expression in the small intestine of mouse

**Figure 6:**
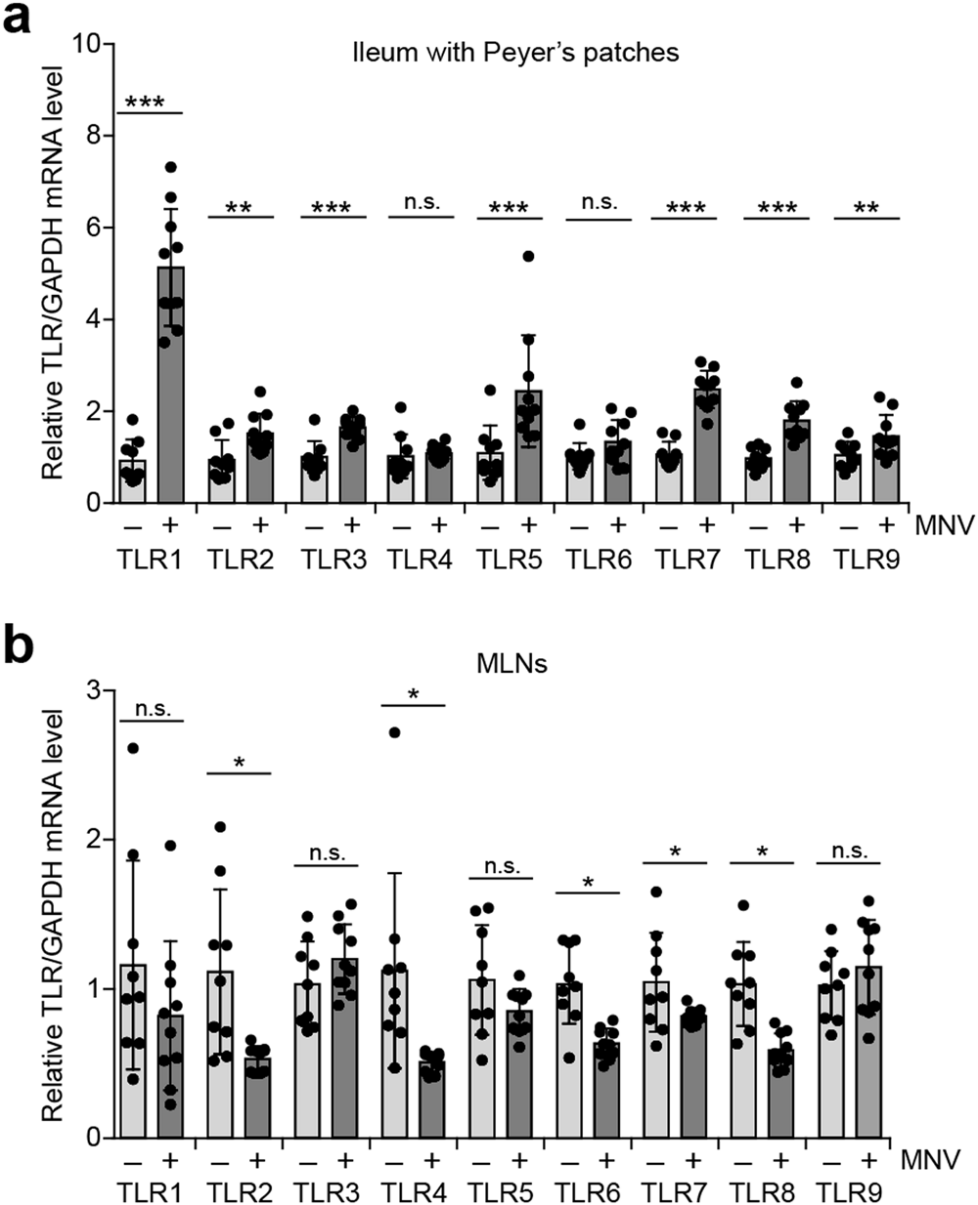
Impact of norovirus infection on TLR expression in mice. The ileum with PPs (**a**) and the MLNs (**b**) from BALB/c mice (6 weeks old, *n* = 9–10 per group) infected with MNV-1 as in Fig. 5 were retrieved 2 days after viral infection and expression of TLR mRNA was quantified as described in Fig. 2f. Data are represented as mean ± SD. *P < 0.05; **P < 0.01; ***P < 0.001; n.s., not significant; by two-tailed unpaired Student’s *t*-test.

Given the possibility of crosstalk between TLRs, if expression of TLRs in the ileum was altered in response to norovirus infection, TLR4 response to γ-PGA would also be affected. The ileum with PPs of norovirus-infected mice showed no significant increase in TLR4 expression. Expression levels of TLR2, TLR3, TLR6, TLR8, and TLR9 were slightly increased. Though the increase was less than 2-fold, it was statistically significant (Fig. 6a). The expression of TLR1, which recognizes peptidoglycan and lipoproteins^43,44^, and TLR5, which recognizes bacterial flagellin^45^ was upregulated by ~5-fold and 2.5-fold, respectively, suggesting the possibility of crosstalk between TLR signaling through some of these upregulated TLRs. This might have contributed to the amplification of γ-PGA-mediated TLR4 signaling *in vivo* as several TLR downstream signaling pathways share transcription factors such as NF-κB and IRF-3^35^, used for type I IFN production. In MLNs, expression levels of TLR2, TLR4, TLR6, TLR7, and TLR8 were slightly decreased (less than 2-fold) (Fig. 6b). These results suggest differentially altered TLR expression profile for norovirus infection in the ileum with PPs and MLNs *in vivo*. Interestingly, this expression profile of TLRs was different from that seen in virus-infected RAW264.7 cells (Fig. 2F), suggesting the involvement of commensal microbiota in the regulation of TLR expression in the ileum, in the context of norovirus infection in mice.

Thus, in the ileum with PPs, where villi responded to γ-PGA and induced IFN-β expression, level of TLR4 remained unchanged although expression of other TLRs was differentially affected by norovirus infection. It remains to be determined whether γ-PGA’s antiviral activity against norovirus was potentiated by the amplification of TLR4 signaling through sensitization of other upregulated TLRs in the ileum upon norovirus infection.

## Discussion

Type I IFN is the primary cytokine that is induced rapidly in response to viral infections. It amplifies immune responses through the IFN-signaling cascade^24,25,46^. However, its antiviral effect in the intestinal tract is less clear. In the present study, we investigated the ability of orally administered γ-PGA to induce an innate immune response and to mount an antiviral state in the ileum. Our results indicate induction of IFN-β production without elevating the level of proinflammatory cytokine by γ-PGA, a non-LPS TLR4 ligand, thereby predisposing the ileum to develop a robust antiviral innate immunity against norovirus.

Our results demonstrate induction of type I IFN production primarily by the activation of TRIF-dependent pathway in mice following multiple peroral administration of γ-PGA. Previous studies have reported the primary activation of MyD88-dependent pathway by LPS in eliciting the secretion of numerous proinflammatory cytokines^1–4,8^. As a TLR4 ligand, γ-PGA is distinct from LPS in selectively increasing the serum IFN-β titers, but not of other inflammatory cytokines, namely IL-1α/β, IL-6, and TNF-α. This difference in the cytokine profile induced by γ-PGA and LPS suggests that a regulatory network different from that of LPS should control γ-PGA-mediated TLR4 activation. It remains unclear whether other non-LPS TLR4 ligands including endogenous TLR4 agonists such as heat shock proteins and cholesteryl ester hydroperoxides^8^ upon being sensed by the same receptor also behave differently than LPS in terms of the cytokine profile. Another unexpected finding was that the elevated serum IFN-β levels in mice after multiple oral administrations of γ-PGA (once daily for three days) neither increased nor decreased following two additional oral administrations. These results suggest the absence of tolerance phenomena with γ-PGA, which is normally seen with endotoxin, or LPS, wherein, innate immune cells pre-exposed to LPS become refractory to subsequent challenges^47^. Based on the results of our present study, γ-PGA seems to be a new class of TLR4 agonists that selectively activates the TRIF-dependent pathway.

IECs are normally non-responsive to TLR4 ligands such as LPS because of low-level expression of TLR4 receptors to prevent undesirable inflammation in response to gut microbiomes or pathogens in the GI tract. Minimal TLR4 expression was seen in normal IECs and the lamina propria rich in scattered inflammatory cells in human biopsies^48^. Although not thoroughly characterized, a previous study had indicated the expression of TLR4 as well as other TLRs (TLR1, TLR2, TLR3, TLR5, and TLR9) in the enterocytes of small intestine^49^. By immunohistochemistry, we observed no prominent TLR4-staining patterns on apical or basolateral surfaces of IECs; TLR4-positive cells were instead localized in the lamina propria of villi and the PPs in the ileum. Thus, our results together with previous findings provide evidence of TLR4 expression in the ileum. However, its fine localization are yet to be well defined. Low but detectable level of TLR4 expression explains its role at the distal ileum that is continuously exposed to microbes and their products transferred from colon^50^. This renders the ileum or IECs hyposensitive to PAMPs including LPS and other TLR agonists, thereby preventing aberrant production of proinflammatory cytokines. In this regard, induction of IFN-β in *ex vivo* cultures of mouse ileum and in mice receiving γ-PGA perorally was surprising, particularly in the absence of upregulation of TLR4 expression by γ-PGA in RAW264.7 cells and in mouse ileum with PPs.

TRIF-dependent pathway of TLR4 signaling is activated in endosomes upon internalization of TLR4-ligand complexes^1–4^. Accordingly, in the present study, γ-PGA delivered orally seemed to be internalized into IECs or transverses the tight junctions between enterocytes to be internalized into the sentinel cells in the lamina propria to induce IFN-β production. Thus, we further investigated as to how γ-PGA reached a site where functional interaction with TLR4 occurred in the ileum. The first barrier for the entry of γ-PGA was the mucus layer, where access of γ-PGA to the small intestine is more likely to occur as it is covered by a thinner mucus layer compared to the colon^51^. For induction of IFN production, γ-PGA needs to be sensed by TLR4 in and/or on villi. Immunostaining revealed lack of overlapping between TLR4 staining and villin, a cytoskeletal protein specific to villi in the ileum, indicating a barely detectable level of TLR4 at the apical site of IECs. We therefore hypothesized a model in which γ-PGA transverses the tight junctions between enterocytes to be sensed by sentinel cells such as dendritic cells within villi or is directly uptaken by transepithelial dendrites (Supplementary Fig. 5). Expression of IFN-β mRNA was significantly enhanced solely in the villi with lack of noticeable IFN-β induction in PPs. Absence of response to γ-PGA in PPs resulting in lack of inflammation was consistent with the previous findings suggesting anergy of intestinal macrophages. Anergy was mediated by their inability to activate NF-κB, and low-level expression of TLR4 and its accessory factors CD14 and MD2^52^.

We previously showed that activation of TLR4 signaling by γ-PGA leading to induction of IFN was dependent on TLR4-associated accessary factors CD14 and MD2^6^. In the present study, results from Q-dot-conjugated γ-PGA confirm the necessity of CD14 and MD2 for sensing γ-PGA. Dependence on CD14 in MyD88-dependent pathway-mediated induction of TNF-α was marginal in LPS-stimulated macrophages^53,54^. However, our results indicate the absolute requirement of CD14 and its prominent role over MD2 in sensing γ-PGA. Our findings suggesting a substantial stimulatory effect of γ-PGA in the lamina propria raises interest on the possibility of differential expression of MD2, CD14, and TLR4 in macrophages and dendritic cells in the lamina propria and PPs. Currently, requirement of these accessory factors in sensing various non-LPS TLR4 ligands remains largely unknown. Irrespective of the mechanisms involved in γ-PGA entry and sensing by TLR4-positive cells in the ileum, both, results from *ex vivo* culture indicating stimulation of the ileum with γ-PGA and the serum cytokine profile of mice receiving peroral γ-PGA, provide clues as to how γ-PGA activates the TRIF-dependent TLR4 signaling pathway in the ileum.

We further investigated the impact of norovirus infection on TLR4-mediated induction of IFN-β by γ-PGA. Our results suggest lack of significant alteration of TLR4 expression in norovirus-infected mice. Thus, responsiveness of TLR4 to γ-PGA was unlikely to be elevated upon norovirus infection. However, norovirus infection led to a ~2-fold increase in the expression of most TLRs, except TLR4 and TLR6 (Fig. 6a). TLR1 level was increased ~5-fold. Upregulated TLR expression could be in part due to the infiltration of leukocytes to the infected ileum. We envision that TLR4 signaling activation by γ-PGA might be potentiated through crosstalks with other upregulated TLRs. Among the upregulated TLRs, the endosomal TLRs including TLR3, TLR7, TLR8, and TLR9 may influence the sensitivity of TLR4 to γ-PGA early during norovirus infection. Currently, their involvement (except TLR3) in innate immune response to norovirus infection remains unknown^24,55^. Although norovirus infection did not affect the expression of TLR4 in the ileum, TLR responses to colon-resident microbiota and/or PAMPs derived from them might influence the downstream cascade of TLR4 signaling. Thus, induction of IFN-β by γ-PGA is likely to be modulated by commensal microbiome in the large intestine adjacent to the ileum.

Type I IFN plays a major role in clearing mouse norovirus^24–26^. It is effective in limiting norovirus replication in human norovirus sub-genomic replicon culture system^17^. In spite of the well-established antiviral potency of type I IFN against noroviruses, IFN therapy is not translated into clinical practice because subcutaneous or intramuscular injection of IFN-α/β is associated with various side effects^56,57^. Our results, demonstrating the prophylactic antiviral activity of γ-PGA in mice using MNV-1 as a model for enteric viruses infecting the small intestine, accentuates its potential use as a safe and prophylactic candidate for the treatment of acute norovirus infection. Although norovirus infection is typically limited to an acute infection that lasts for 2–3 days in most healthy subjects^58^, it is associated with significant clinical outcomes with ~200,000 deaths related with gastroenteritis in children under 5 years of age^59^. In young children, the elderly, and immunocompromised patients for whom IFN might not be the best treatment option, γ-PGA can be used as an alternative prophylactic antiviral agent for HuNoV. It is important to note that unlike the MNV replicating mainly in macrophages and dendritic cells^25^, HuNoV, which was shown to replicate in B cells^36^, also targets IECs^60–62^. Therefore, the IFN produced by the sentinel cells in the lamina propria of villi upon sending the γ-PGA might restrict HuNoV infection more effectively at the early stage of infection by acting on nearby IECs through paracrine signaling.

In summary, our results demonstrate that oral administration of γ-PGA induces IFN-β production in the ileum without upregulating the expression of TLR4 and without eliciting the production of inflammatory cytokines. The specific IFN-β induction feature of γ-PGA highlights its potential application as a prophylactic antiviral agent for noroviruses and possibly other enteric viruses.

## Methods

### Cell culture

The murine macrophage cell line RAW264.7 and human embryonic kidney 293 T (HEK293T) cell line were purchased from American Type Culture Collection (ATCC; Rockville, MD, USA) and cultured in Dulbecco’s modified Eagle’s medium (DMEM) supplemented with 10% fetal bovine serum (FBS), 100 U/ml penicillin, and 100 µg/mL streptomycin. The HG23 cell line stably harboring a HuNoV (Norwalk virus) replicon^17^ was cultured in DMEM supplemented with 10% FBS, 2 mM l-glutamine, 100 U/ml penicillin, 100 µg/ml streptomycin, and 0.1 mM nonessential amino acids in the presence of 1 mg/ml neomycin (G418 sulfate). Cells were maintained at 37 °C in a 5% CO_2_ humidified incubator.

### Reagents and transfection

Endotoxin-free γ-PGAs with different average molecular masses (500- and 2,000-kDa), which were prepared as described previously^60^, were provided by BioLeaders (Daejeon, Korea). The plasmids expressing human TLR4, CD14, and MD2 were described^61,62^. In transient expression experiments, empty vector was added to normalize the total amounts of transfected DNA. Plasmids were transfected into cells using Fugene HD transfection reagent (Roche Diagnostics, Indianapolis, IN, USA). Chemically synthesized siRNAs were purchased from Bioneer (Daejeon, Korea). The siRNA used are as follows: siTLR4 [siRNA targeting mouse TLR4, 5′-GAAUUGUAUCGCCUUCUUAdTdT-3′ (passenger stand) and 5′-UAAGAAGGCGAUACAAUUCdTdT-3′ (guide strand)] and siCtrl [control siRNA targeting GFP, 5′-CUCGCCGGACACGCUGAACUU-3′ (passenger stand) and 5′-GUUCAGCGUGUCCGGCGAGUU-3′ (guide strand)]. siRNAs were transfected into RAW264.7 cells using the ND98 lipidoid as a transfection reagent^63^. Mouse polyclonal anti-IRF-3 (FL-425), rabbit monoclonal anti-phospho-IRF-3(Ser396), and rabbit polyclonal anti-β-actin antibody were purchased from Cell Signaling Technology (Beverly, MA, USA). Anti-norovirus capsid protein VP1 antibody was purchased from Abcam (Cambridge, UK). Anti-TLR2 (H-175), anti-TLR3 (N-14), anti-TLR4 (25), and anti-villin antibodies were purchased from Santa Cruz Biotechnology (Santa Cruz, CA, USA). Anti-α-tubulin antibody was purchased from Calbiochem (La Jolla, CA, USA).

### Purification of MNV and infection

MNV-1.CW1^64^ was kindly provided by Herbert W. Virgin (Washington University School of Medicine, St. Louis, MO, USA) and propagated in RAW264.7 cells to generate a viral stock. Briefly, RAW264.7 cells were infected with MNV at a multiplicity of infection (MOI) of 0.5 in a serum-free medium and incubated for 1 h. Following washing, infected cells were further cultivated in fresh complete media for 2 days. Viral stock was prepared by centrifuging harvested media at 450 × *g* for 10 min. Intracellular virus particles recovered by three freeze/thaw cycles were harvested by centrifugation at 3,000 × *g* for 10 min. Virus samples were then concentrated using an Amicon Ultra centrifugal filter unit with a molecular weight cutoff of 50 kDa (Merck Millipore, Billerica, MA, USA) and stored at −80 °C until further use as crude MNV preparation. Purification of MNV was performed as previously described with modifications^65^. Briefly, crude MNV was pelleted by ultracentrifugation at 150,000 × *g* for 1 h at 4 °C. The pellet was gently suspended in a suspension buffer (50 mM Tris-HCl, pH 7.5, 100 mM NaCl) at 4 °C and pelleted again by ultracentrifugation. The resultant pellet was dissolved in suspension buffer and was subjected to sucrose density gradient (10–60%) centrifugation at 100,000 × *g* for 150 min at 4 °C to collect the MNV-containing fractions that were identified by SDS- polyacrylamide gel electrophoresis (PAGE) followed by immunoblotting for the VP1 capsid protein. The fractions were subjected to ultracentrifugation as described above to obtain purified virus particles. These purified virus particles were suspended in PBS and used for virus entry experiments.

### Plaque-forming assay

Plaque-forming assay was performed as described previously^66^. Briefly, RAW264.7 cells seeded in 6-well plates (2 × 10^6^ cells/well) were cultured overnight and infected with norovirus in serum-free DMEM. After 1 h of infection, cells were washed to remove the inoculum and were overlaid by SeaPlaque Agarose (1% w/v; Lonza, Rockland, ME, USA) in complete DMEM with high glucose. Following 2 or 3 days of culture, plaques were visualized by staining the monolayer cells with neutral red.

### Cell viability

RAW264.7 cells seeded in 96-well plates (2 × 10^4^ cells/well) were grown overnight and treated with various concentrations of γ-PGA for 48 h. Cell viability was assessed by an MTS [3-(4,5-dimethylthiazol-2-yl)-2,5-diphenyltetrazolium bromide] assay as described previously^67^.

### RNA extraction and RT-qPCR

For total RNA extraction from mouse ileum, three pieces (1-cm-long segment) of the ileum with Peyer’s patches (~3-mm each in length) were retrieved from the distal ileum (distal 1/3 part of the ileum), opened, cleaned by washing with PBS to remove fecal matter, and homogenized in 1-ml PBS. Similarly, total RNA was recovered from MLNs. Total RNA from cells or culture media was extracted with TRIzol or TRIzol LS reagent (Invitrogen, Carlsbad, CA, USA), respectively. Norwalk virus and MNV-1 RNA copy number was determined by RT-qPCR as described previously^68,69^. Quantification of mRNA was carried out by RT-qPCR using SYBR Premix ExTaq (Takara, Japan). The specific primer sets used are listed in Supplementary Table 1. Specific primers for human IFN-β and glyceraldehyde 3-phosphate dehydrogenase (GAPDH) were as described previously^70^. The expression levels of all the target genes normalized with the expression level of GAPDH mRNA were quantified using the ΔΔC_t_ method^71^.

### Immunoblot analyses

Cells were lysed in lysis buffer (50 mM Tris-HCl, pH 8.0, 150 mM NaCl, 1 mM EDTA, 1% Nonidet P-40) supplemented with EDTA-free protease inhibitor cocktail (Roche Diagnostics) by incubating on ice for 20 min. Aliquots of cleared cell lysates from the same samples were resolved by SDS-PAGE, transferred onto nitrocellulose membranes, and processed in parallel for immunoblotting analyses for multiple proteins. Membranes were immunoblotted with specific antibodies and the bound antibodies were detected with an enhanced chemiluminescence kit (GE Healthcare Life Sciences, Piscataway, NJ, USA).

### Transmission electron microscopy

Purified MNV particles absorbed onto carbon-coated grids were negatively stained with uranyl acetate. The stained samples were examined under a Tecnai G2 Spirit TWIN transmission electron microscope (FEI Company, OR, USA) at the Division of Electron Microscopic Research, Korea Basic Science Institute (KBSI; Daejeon, Korea).

### Analyses of cellular entry of γ-PGA and MNV

For microscopic analysis, both, γ-PGA and purified MNV were conjugated with biotin on the exposed amine group using Sulfo-NHS-LC-Biotin (EZ-Link; Pierce, Rockford, IL, USA) according to the manufacturer’s protocol. Biotinylated γ-PGA and MNV-1 were detected using Qdot 625 streptavidin conjugate (Invitrogen).

### Immunohistochemistry and confocal microscopy

Intestinal tissues of mice were embedded in a frozen section compound (Surgipath FSC22, Leica Microsystems, Wetzlar, Germany). Thin sections (10 µm) were fixed in 4% paraformaldehyde, blocked in 1% BSA in PBS, and incubated for 2 h at room temperature with an anti-TLR4 mouse monoclonal antibody or an anti-villin rabbit polyclonal antibody with gentle rocking. After washing 3 times with PBS, appropriate fluorescent-conjugated secondary antibodies were used for visualizing the antigen. Nuclei were visualized by staining with 1 μM 4′,6′-diaamidino-2-phenylindole (DAPI) in PBS for 10 min. Confocal images were acquired on a ZEISS LSM 880 confocal laser scanning microscope (Carl Zeiss, Oberkochen, Germany). For z-stack analysis, images were obtained along the z-axis at 0.3-μm intervals and subjected to three-dimensional analysis using ZEN software (Blue edition, 2.3, Carl Zeiss).

### Enzyme-linked immunosorbent assay (ELISA)

The serum levels of IFN-α and β were measured using the VeriKine mouse IFN-α and β ELISA kit (PBL Interferon Source, Piscataway, NJ, USA). The detection limit of mouse IFN-α and β ELISAs were 12.5 and 15.6 pg/ml, respectively. Serum levels of other cytokines (IL-1α, IL-1β, IL-2, IL-6, IL-10, TNF-α, and IFN-γ) were quantified using Luminex multiplex detection assay using antibody-coated magnetic beads (R&D Systems, Minneapolis, MN, USA).

### *Ex vivo* culture of small intestinal tissues of mouse

For e*x vivo* culture of small intestinal tissues of mouse, ileum was isolated and cultured as described previously^72^ with slight modifications. Briefly, distal ileum segments (1 cm in length) with or without Peyer’s patches were collected, gently opened with a sterilized operating scissors, washed five times with PBS supplemented with 10% penicillin/streptomycin, and transferred to 12-well plates for further incubation in complete DMEM.

### *In vivo* antiviral activity of γ-PGA

BALB/c mice (6 weeks old, *n* = 10 per group) were orally administered with γ-PGA in 100-µl saline once a day for 1, 3, or 5 days at a dose of 50 mg/kg body weight. Four hours after the last γ-PGA administration, total blood was collected from the abdominal vein of mice for cytokine analyses using ELISA. To test the antiviral efficacy of γ-PGA, BALB/c mice (6 weeks old, *n* = 7 per group) were orally administered with γ-PGA (50 mg/kg body weight) in 100 µl saline once a day for 5 days. This was followed by peroral inoculation with MNV (1 × 10^7^ PFU/100 µL saline) 12 h after the last γ-PGA administration at day 5. γ-PGA was given once daily for another two days. Two days after inoculation, the distal ileum and MLNs were retrieved from mice for analyses of intracellular viral RNA and infectious virus titers. Homogenized tissues were subjected to centrifugation at 3,000 × *g* for 10 min to recover supernatants, which were then passed through a 0.20-µm pore-size membrane filter. The filtrates were used for quantification of virus titers using a plaque-forming assay.

### Animal experiments

All animal experiments were performed in accordance with the Korean Food and Drug Administration guidelines. Protocols were reviewed and approved by the Institutional Animal Care and Use Committee of the Yonsei Laboratory Animal Research Center (Permit No: IACUC-A-201409-280-02; continued on 201502-153-01). For collection of serum and tissues, mice were anesthetized with intraperitoneal injection of tribromoethanol (Avertin). At the termination of the experiment, all mice were euthanized by CO_2_ inhalation.

### Statistical analysis

Statistical analysis was performed using GraphPad Prism 6 (Graphpad Prism Software Inc., La Jolla, CA, USA). Results are presented as the mean ± standard deviation (s.d) from at least three independent experiments, unless otherwise stated. Specific tests used to determine statistical difference are noted in each figure legend. Differences between groups were considered statistically significant if *P < 0.05.

### Data availability

The authors declare that all other relevant data are available from the authors upon request.

## Electronic supplementary material

Supplementary Information
