# Supplementary material for: Prophylactic efficacy of orally administered Bacillus poly-γ-glutamic acid, a non-LPS TLR4 ligand, against norovirus infection in mice
Source: Sci Rep. 2018 Jun 6;8:8667. doi: 10.1038/s41598-018-26935-y (PMC5989232; doi:10.1038/s41598-018-26935-y)
Supplement: Supplementary file 1 — Supplementary Information [file 41598_2018_26935_MOESM1_ESM.pdf]

Supplementary Information

Prophylactic efficacy of orally administered *Bacillus* poly-γ-glutamic acid, a non-LPS TLR4 ligand, against norovirus infection in mice

Wooseong Lee, Minwoo Kim, Seung-Hoon Lee, Hae-Gwang Jung & Jong-Won Oh*

Contents:

1. Supplementary Figures 1–6
2. Supplementary Table 1

Correspondence should be addressed to J.-W.O. (jwoh@yonsei.ac.kr).

Supplementary Figures

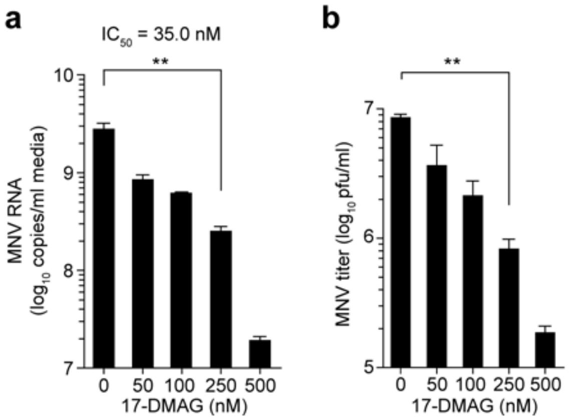

Supplementary Figure 1. Antiviral potency of 17-DMAG, an Hsp90 inhibitor, against norovirus. RAW264.7 cells infected with MNV-1 at a MOI of 0.05 were treated with increasing concentrations of 17-DMAG. Following 24 h of incubation, intracellular genome titer (**a**) and infectious virus titer in culture media (**b**) were assessed by RT-qPCR and plaque-forming assay, respectively. Error bars are standard deviations of the mean values of results from three independent experiments each involving triplicate assays. *P* values were calculated using a two-tailed unpaired Student's *t*-test. $^{**}P < 0.01$. The IC_{50} value was calculated using the SigmaPlot software.

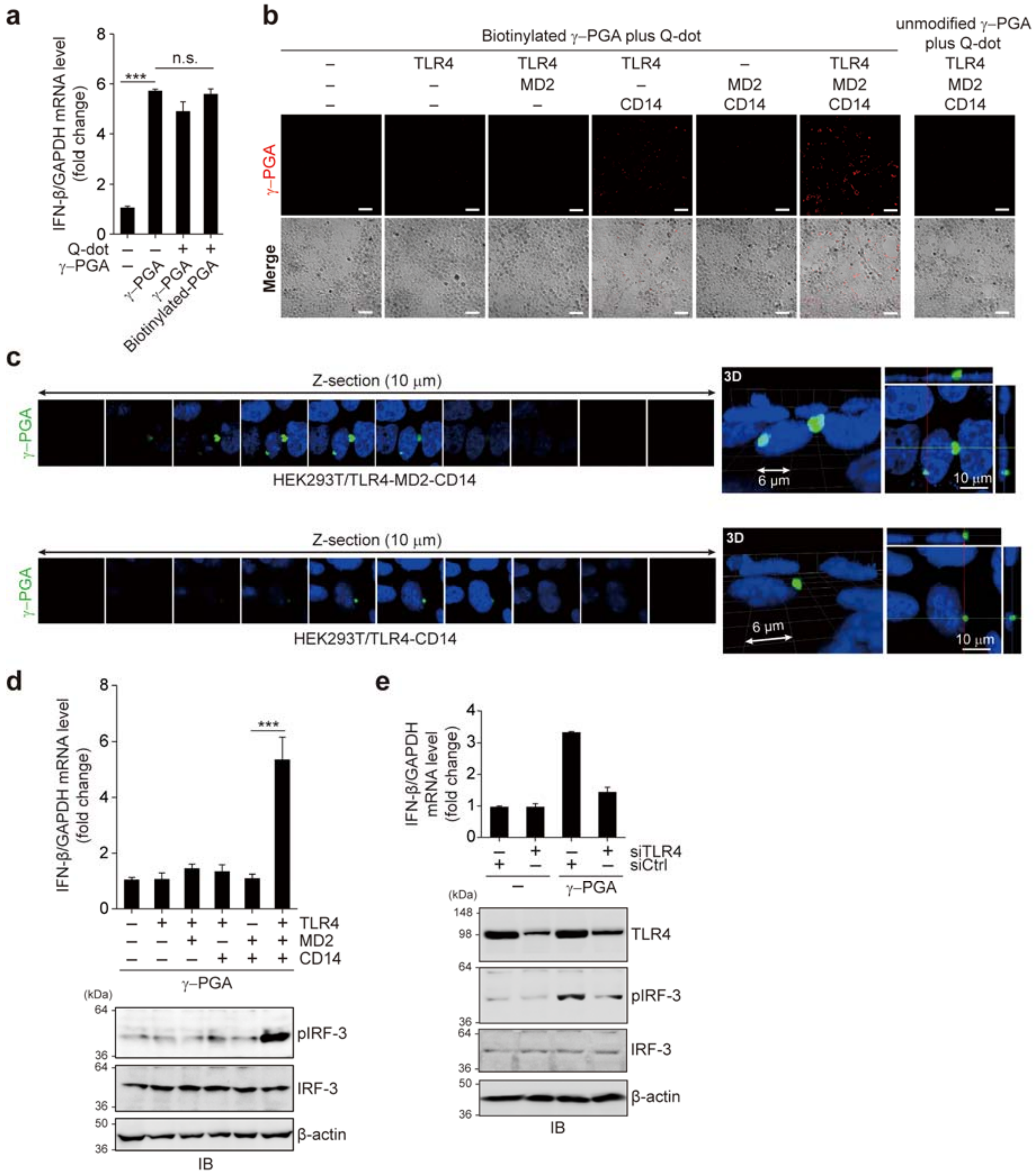

Supplementary Figure 2. MD2 and CD14-dependent sensing of γ-PGA by TLR4 is required for induction of IFN-β expression and IRF-3 activation. (**a**) Induction of IFN-β mRNA expression by biotinylated γ-PGA bound to streptavidin-coated Q-dots. Unmodified or biotinylated γ-PGA (2,000-kDa), in free form or bound to streptavidin-coated Q-dots, was incubated with HEK293T cells transiently expressing TLR4, CD14, and MD2. Following 24 h of incubation, IFN-β mRNA levels (normalized to GAPDH) were determined by RT-qPCR. ***$P < 0.001$; n.s., not significant; by unpaired Student *t*-test. (**b**) Both MD2 and CD14 are required to facilitate γ-PGA sensing by TLR4. HEK293T cells transiently expressing TLR4 with or without MD2 and/or CD14 were incubated with unmodified or biotinylated γ-PGA mixed with streptavidin-coated Q-dots Following 2 h of incubation, γ-PGA was visualized by confocal microscopy. Scale bar, 100 μm. (**c**) γ-PGA sensing and internalization require both TLR4 and CD14. Confocal microscopy of the internalized γ-PGA in HEK293T cells transiently expressing TLR4, CD14, and MD2. Confocal images taken after 6 h incubation

with the Q-dot-bound biotinylated γ-PGA are presented as x-y (en face) and z-stack images. Nuclei were counter-stained with DAPI dye. Scale bar, 10 μm. (**d**) Induction of IFN-β expression and IRF-3 activation in HEK293T cells transfected with the indicated expression vectors and stimulated with γ-PGA. IFN-β mRNA levels were determined by RT-qPCR as described in (**a**). Immunoblot analysis for the indicated proteins was performed using total cell lysates at 24 h post-stimulation with γ-PGA (100 nM). (**e**) Impact of TLR4 depletion by RNAi on induction of IFN-β expression and IRF3 activation by γ-PGA. RAW264.7 cells transfected with siTLR4 or siCtrl (40 nM) for 24 h were left untreated or stimulated with γ-PGA (100 nM) for 24 h prior to quantification of IFN-β mRNA levels and immunoblot analysis.

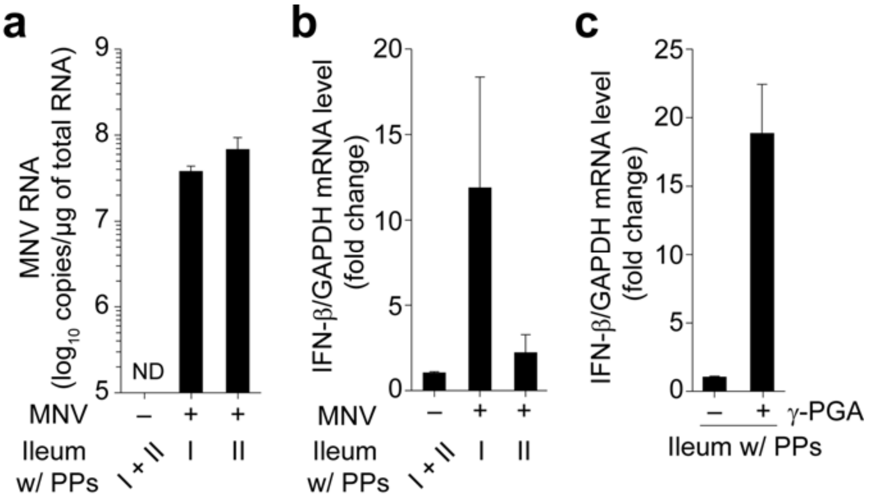

Supplementary Figure 3. Induction of IFN-β expression by MNV and γ-PGA in mouse ileum. One milliliter of MNV (10^7 PFU) (**a**,**b**) or γ-PGA (100 nM) (**c**) was infused directly into mouse ileum after knotting the upper part of the duodenum and cleaning out stool through the ileal terminal ends by washing once with 10 ml of PBS. Following 2 h of incubation, ileum with PPs was collected and incubated for 6 h in complete culture media, prior to quantification of IFN-β mRNA and MNV genomic RNA by RT-qPCR. I and II indicate the compartments of ileum with PPs as depicted in Fig. 3a.

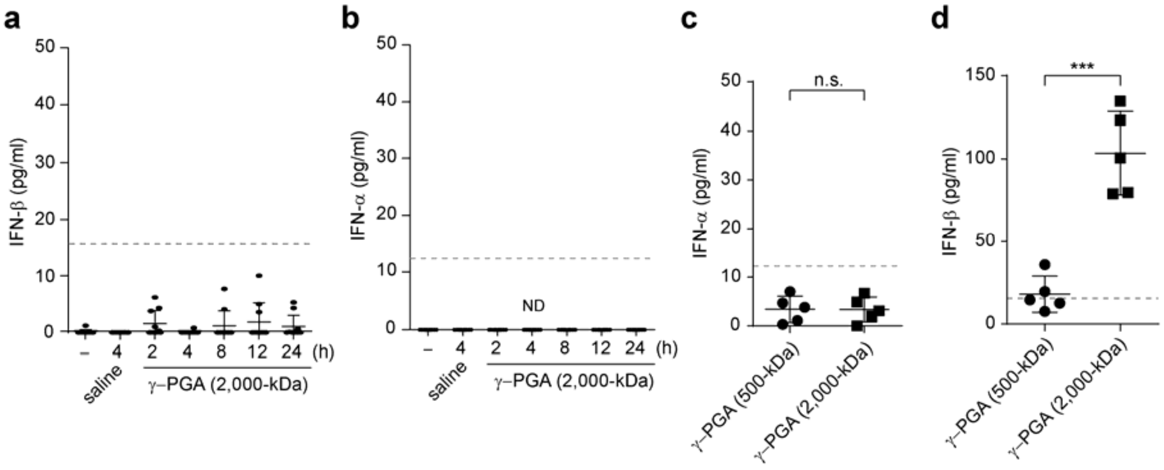

Supplementary Figure 4. Induction of IFN-β expression by multiple oral administrations of γ-PGA in mice. (**a**,**b**) BALB/c mice (six weeks old, n = 10 per group) were orally administered with 2,000-kDa γ-PGA (50 mg/kg of body weight) in 100-μl saline to monitor the serum levels of IFN-β and IFN-α by multiplex ELISAs at the indicated time points. (**c**,**d**) BALB/c mice (six weeks old, n = 5 per group) were given 500-kDa or 2,000-kDa γ-PGA perorally as described above once daily for five days. Following 4 h after the last administration, serum IFN-α and IFN-β levels were assessed by ELISAs. The dotted line shows the limit of detection. ***$P < 0.001$; n.s., not significant; by unpaired Student's *t*-test. ND, not detected.

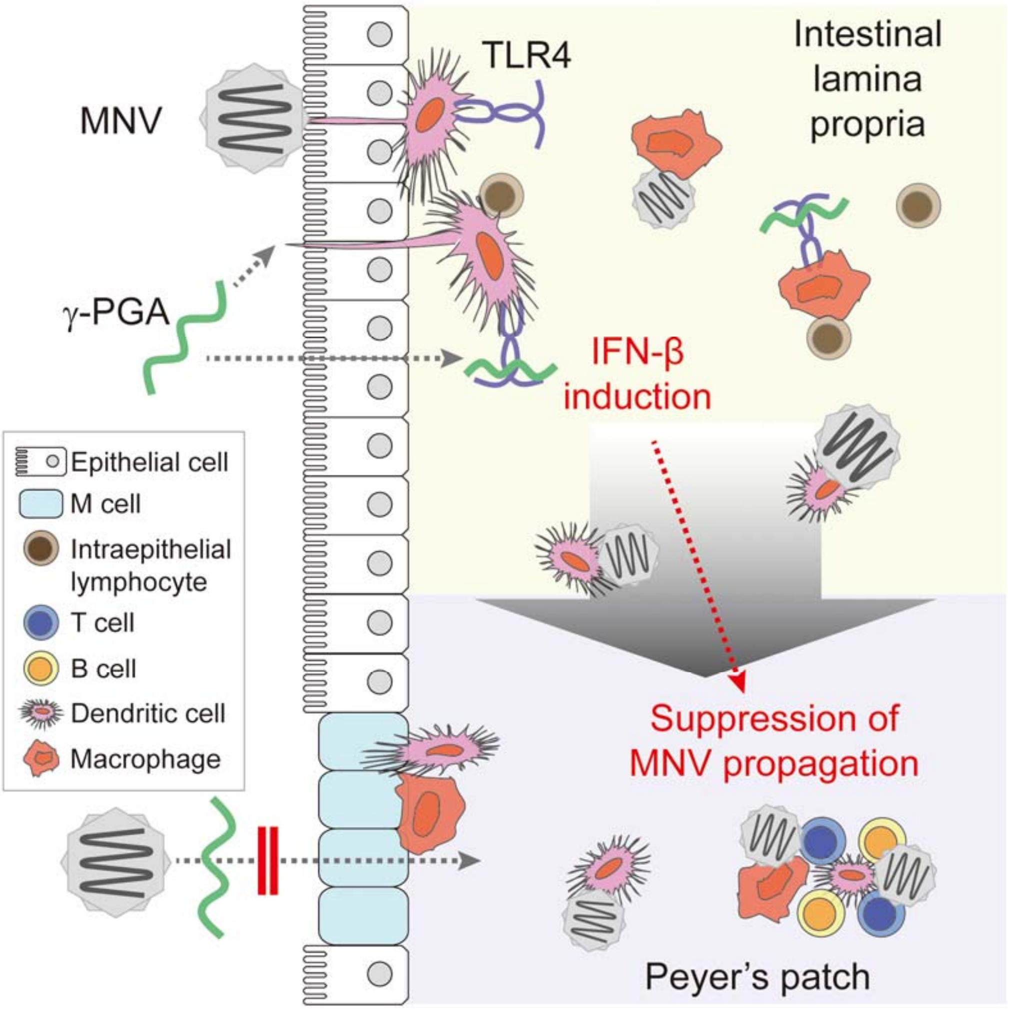

Supplementary Figure 5. Model for TLR4-mediated IFN-β production by γ-PGA in the small intestine to mount innate antiviral immunity against norovirus. γ-PGA enters into intestinal lamina propria primarily through the villi to induce IFN-β production by dendritic cells and macrophages via the TLR4-TRIF signaling pathway. γ-PGA exerts its prophylactic antiviral activity by predisposing the ileum to mount a rapid innate immune response against norovirus infection and through its ability to inhibit viral entry, thereby enhancing the clearance of norovirus.

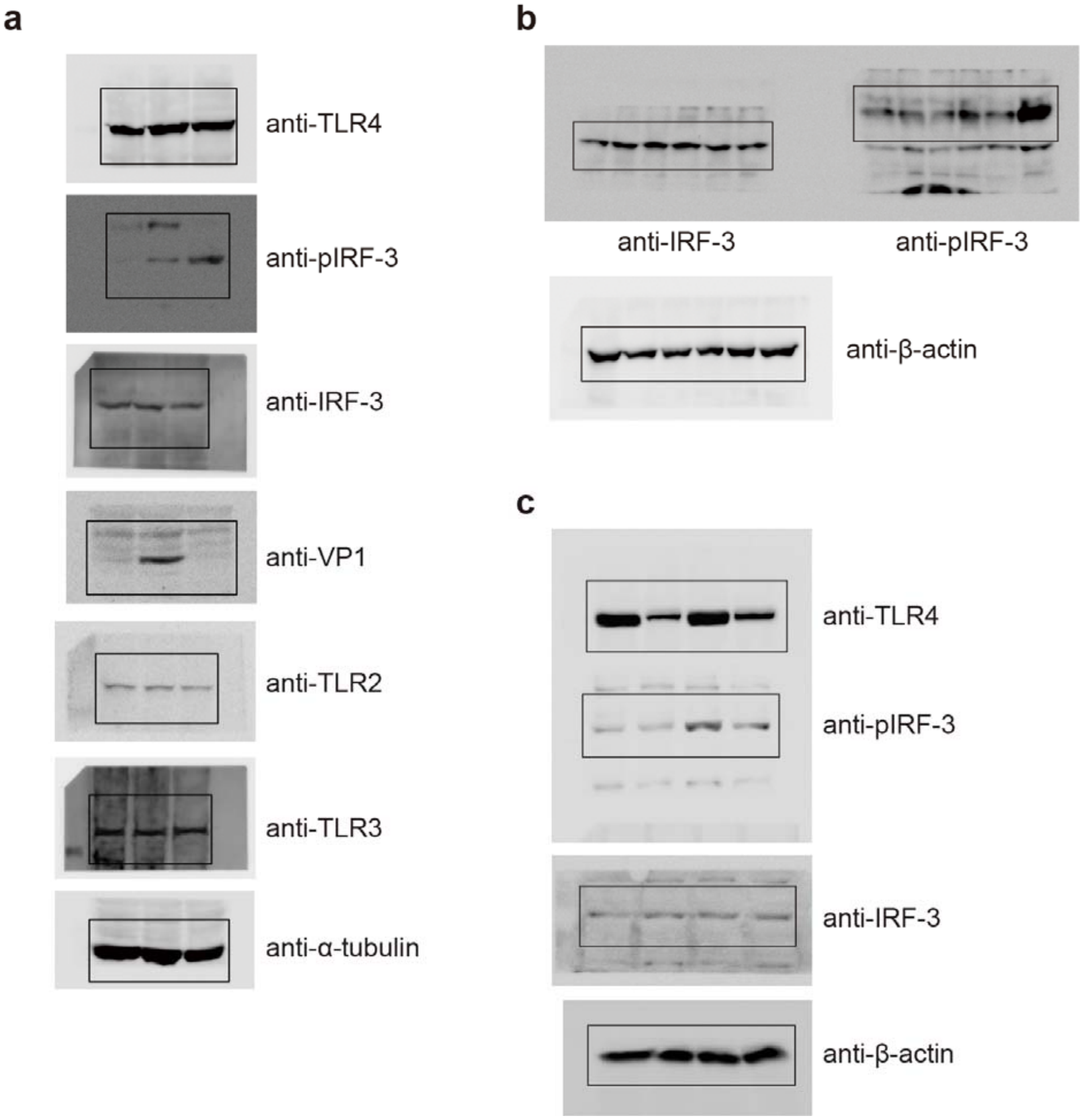

Supplementary Figure 6. Uncropped images of immunoblots. (**a**) For Fig. 2g. (**b**) For Supplementary Fig. 2d. (**c**) For Supplementary Fig. 2e.

Supplementary Table

Supplementary Table 1. Primers used for quantification of mRNA and norovirus genome levels by RT-qPCR

Gene	Primer set	Sequence (5′-3′)[a]	Reference
MNV-1	Forward	CACGCCACCGATCTGTTCTG	64
	Reverse	GCGCTGCGCCATCACTC	
NoV-GI	Forward	CGYTGGATGCGNTTYCATGA[a]	63
	Reverse	CTTAGACGCCATCATCATTYAC[a]	
mIFN-β	Forward	AACAGGTGGATCCTCCACGCTGCG	68
	Reverse	GTGGAGAGCAGTTGAGGACATCTCC	
TLR1	Forward	TCTTCGGCACGTTAGCACTG	69
	Reverse	CCAAACCGATCGTAGTGCTGA	
TLR2	Forward	GCCACCATTTCCACGGACT	69
	Reverse	GGCTTCCTCTTGGCCTGG	
TLR3	Forward	GGTCCCCAGCCTTCAAAGAC	69
	Reverse	ACGAAGAGGGCGGAAAGGT	
TLR4	Forward	AGAAATTCCTGCAGTGGGTCA	69
	Reverse	TCTCTACAGGTGTTGCACATGTCA	
TLR5	Forward	ATGGCATGTCAACTTGACTT	69
	Reverse	GATCCTAAGATTGGGCAGGT	
TLR6	Forward	TCATCTCAGCAAACACCGAGTATAGCG	69
	Reverse	CAACCTTATTGAATGTGACCCTCCAGC	
TLR7	Forward	GTACCAAGAGGCTGCAGATTAGAC	69
	Reverse	AGCCTCAAGGCTCAGAAGATG	
TLR8	Forward	GAAGACGATTTCGCCAAGAG	69
	Reverse	GAAGCATTTCGAGCATCTCC	
TLR9	Forward	ACTTCGTCCACCTGTCCAA	69
	Reverse	AGGAAGGTTCTGGGCTCAAT	
GAPDH	Forward	GAAGGTGAAGGTCGGAGTC	
	Reverse	GAAGATGGTGATGGGATTTC	

[a] Mixed bases are as follows: Y, C or T; N, any. mIFN-β, mouse IFN-β. NoV-GI, human norovirus genotype I.
